# Nuclear Anxiety Amid the Russian-Ukrainian War 2022 (RUW-22): Descriptive Cross-Sectional Study

**DOI:** 10.3390/ijerph20043551

**Published:** 2023-02-17

**Authors:** Abanoub Riad, Anton Drobov, Muhammad Abdullatif Alkasaby, Aleš Peřina, Michal Koščík

**Affiliations:** 1 Department of Public Health, Faculty of Medicine, Masaryk University, 625 00 Brno, Czech Republic; 2 Centre for Global Mental Health, London School of Hygiene & Tropical Medicine, Keppel St., London WC1E 7HT, UK

**Keywords:** anxiety, armed conflicts, Czech Republic, depression, nuclear power plants, patient health questionnaire, radioactive hazard release

## Abstract

Nuclear anxiety, which refers to the fear of nuclear war and its consequences, is expected to increase amid the Russian–Ukrainian War of 2022 (RUW-22). This study aimed to evaluate the prevalence of nuclear anxiety and its associated variables among university students in the Czech Republic during the first weeks of RUW-22. A cross-sectional survey-based study was carried out from March–April 2022, utilizing a digital self-administered questionnaire (SAQ) to collect data from the target population. The SAQ consisted of multiple-choice items inquiring about demographic characteristics; generalized anxiety symptoms using generalized anxiety disorder-7 (GAD-7); depressive symptoms using patient health questionnaire-9 (PHQ-9); and attitudes towards civilian uses of nuclear power, and nuclear war-related anxiety. Of the 591 participating students, 67.7% were females, 68.2% were Czech nationals, and 61.8% followed the RUW-22 news at least once daily. The mean GAD-7 score of our participants was 7.86 ± 5.32 (0–21); and their mean PHQ-9 score was 8.66 ± 6.29 (0–27). Regarding the civilian uses of nuclear power, most participants agreed that nuclear power was safe (64.5%), denied being afraid that civilian use of nuclear power might deteriorate their health (79.7%), and thought that public acceptance was important for building new nuclear power plants (56.9%). About 42.1% and 45.5% of the participants reported feeling depressed at the possibility of nuclear war and agreed that the chances that there would be a nuclear war in their lifetime were very high, respectively. When asked about their preparedness measures during the previous four weeks, less than one quarter (23.9%) reported looking for recommendations for protection against nuclear accidents, and less than one-fifth (19.3%) were looking for the nearest bomb shelter. The depression about nuclear war possibility was positively and relatively strongly correlated with the level of “feeling concerned about the RUW-22” (rs = 0.401), and it was moderately correlated with GAD-7 (rs = 0.377) and PHQ-9 (rs = 0.274) scores and weakly correlated with RUW-2-related news-following frequency (rs = 0.196). Within the limitations of the present study, nuclear anxiety was common among Czech university students. Its associated factors may include but are not limited to the female gender; common psychological disorders such as generalized anxiety and depression; RUW-22-related news following-frequency; and the level of “feeling concerned”.

## 1. Introduction

Nuclear anxiety can be defined as “fear of nuclear war and of its consequences” [1]. It was first described by the American anthropologist Margaret Mead, who advocated in the 1960s that this fear should be directed towards the need for peace rather than rallying for apocalyptic armament [2]. The Cold War is seen as an inaugural event for scholarly interest in this topic, as several social scientists attempted to delve into the aetiology and magnitude of this emerging phenomenon. For instance, the Nuclear Attitudes Questionnaire (NAQ) of Newcomb (1986) is a psychometric instrument that consists of 15 items that aim to evaluate nuclear concern, nuclear denial, nuclear support, and fear of the future [3,4]. Among a sample of young adults, females had a significantly higher level of nuclear concern and fear of the future and a lower level of nuclear support and nuclear denial [4]. From a longitudinal perspective, nuclear anxiety, denoted by nuclear concern and nuclear fear, was found to be constantly increasing among young adults in the United States (US) who were surveyed repeatedly during the 1980s [3].

International conflicts are key triggers for nuclear anxiety at individual and community levels; therefore, the era of the Cold War, which is well-known for geopolitical tensions between the Western Bloc and the Eastern Bloc, was characterized by nuclear anxiety [5]. The Cuban Missile Crisis (or the Missile Scare) of 1962 is a perfect example of how mutual threats between the US and the Soviet Union (USSR) led to international instability and anticipated nuclear attacks [6]. On top of that, the Chernobyl disaster that happened in Ukraine during the Cold War is the most catastrophic nuclear event yet, as it released more than 5300 petabecquerels of radioactive materials compared with the second most notable nuclear accident in human history (Fukushima accident), which released only 520 petabecquerels [7].

The Russian–Ukrainian War of 2022 (RUW-22) is unarguably the most tragic event in modern European history since the Second World War (WWII), which engenders financial, social and health burdens that remain beyond estimation [8]. As of 1 July 2022, more than 12 million Ukrainians were forced to flee their homes, with over five million refugees received by neighbouring countries and about seven million internally displaced [9]. As a military conflict, RUW-22 is expected to generate substantial pressure on health systems in Ukraine and neighbouring countries due to the surging needs of the affected communities [10,11]. According to the World Health Organization (WHO), mental health surveillance and support are essential functions of response strategies for public health emergencies, such as infectious disease outbreaks and military conflicts [12,13].

In addition to the anticipated mental health impact of this war on Ukrainian civilians, especially children and young adults, the neighbouring European communities are threatened by the sequela of potential nuclear attacks [14]. One week after the war broke out, the Russian president signalled the possibility of using nuclear weapons during this war [15]. A few days later, while Russian forces were attempting to seize Europe’s largest nuclear power plant in Zaporizhzhia, blazes and explosions within the territory of the power plant were reported [16]. However, nuclear anxiety can expectedly increase in response to the RUW-22; there is a paucity of evidence on its prevalence, associated factors, and association with common mental health disorders, e.g., generalised anxiety disorder and depression.

The outbreak of the RUW-22 was widely apprehended by the European populations, especially those living in Central and Eastern European countries such as the Czech Republic [17], Poland [18], Germany [19], and Italy [20]. The brutal invasion of Russian troops of Ukraine in February 2022 reminded Czech citizens of what happened in August 1968 when Soviet tanks invaded Prague to abort the new progressive socioeconomic movement known as Prague Spring [21,22,23]. The Czech response to the RUW-22 was multifaceted and included the Czech senate’s recognition of RUW-22 as a genocide, calling for accelerated weapons supply to Ukraine, joining NATO forces, and EU sanctions on Russia. It also called for ending the country’s dependence on Russian oil and gas [24].

The present study aimed to explore nuclear anxiety among Czech university students and its associated factors. Additionally, the association between nuclear anxiety and generalised anxiety disorder, depression, level of “feeling concerned about the RUW-22”, and RUW-22 news-following frequency was evaluated.

## 2. Materials and Methods

### 2.1. Design

A descriptive cross-sectional study was conducted between March–April 2022 utilising a self-administered questionnaire that was designed and accessed digitally through KoBoToolbox (Harvard Humanitarian Initiative, Cambridge, MA, USA, 2022) [25]. The Strengthening the Reporting of Observational Studies in Epidemiology (STROBE) guidelines for cross-sectional studies were followed while designing and reporting the present study [26].

### 2.2. Population

As young adults were the target population, Czech university students were approached to participate in this study. Non-random sampling was used as the target participants were invited through the social media accounts of Masaryk University in Brno, e.g., Facebook, Instagram, and Twitter. Additionally, students’ unions and organizations joined forces to promote the survey among their members to increase the response rate.

The pragmatic sample size was computed by Epi-Info^TM^ version 7.2.5 (CDC. Atlanta, GA, USA, 2021), assuming that the target population (N) was ≈300,000, the expected frequency was 50%, the error margin was 5%, the confidence level (CI) was 95%, and the response rate generated by careless or insufficient effort (C/IE) was 10% [27]. Four hundred and twenty-three responses were required.

### 2.3. Instrument

The present study utilised an SAQ which consisted of close-ended questions inquiring about: (i) demographic characteristics, e.g., gender, age, and nationality; (ii) the level of “feeling concerned with the RUW-22 news” assessed by an 11-point hedonic scale; (iii) frequency of the RUW-22 news following; (iv) generalised anxiety symptoms assessed by the Generalized Anxiety Disorder-7 (GAD-7) of Spitzer et al. 2006; (v) depressive symptoms assessed by the Patient Health Questionnaire-9 (PHQ-9) of Kroenke et al. 2001; (vi) attitudes towards civilian usage of nuclear power; and (vii) nuclear-war related anxiety [28,29]. The items of nuclear war-related anxiety were adapted from instruments developed during the Cold War [3,30].

The content validity of the draft SAQ was evaluated by a panel of experts in public health and clinical psychology. The construct validity was verified by confirmatory factor analysis (CFA) which indicated a good model fit (RMSEA: 0.05; CI 95%: 0.048–0.057). Additionally, the test re-test reliability of the proposed SAQ was tested through a group of volunteer students (*n* = 10) who were invited to fill in the questionnaire twice. The proposed SAQ had moderate reliability, denoted by a mean Cohen’s Kappa coefficient of 0.532 ± 0.155. The full psychometric properties of the SAQ were reported previously [17].

### 2.4. Ethics

The principles laid by the Declaration of Helsinki for research involving human subjects and the General Data Protection Regulation (GDPR) of the European Union (EU) were followed while conducting the present study [31,32]. All participants had to provide their informed consent digitally before accessing the SAQ, and they were capable of withdrawing from the study anytime without justification.

### 2.5. Analyses

Initially, the normal distribution of numerical variables, e.g., GAD-7 and PHQ-9 scores, was tested using the Shapiro–Wilk test with a significance level of <0.05. Descriptive statistics were carried out using frequencies (n) and percentages (%) for qualitative variables, e.g., gender and nationality, and means and standard deviations (µ ± SD) for quantitative variables. Chi-squared (χ^2^), Fisher’s exact, Mann–Whitney (U) and Kruskal–Wallis (H) tests were used to test the associations between dependent and independent variables. Additionally, nonparametric correlation using Spearman’s coefficient (*r_s_*) and linear regression were conducted. Inferential tests were executed under the assumptions of a 95% of confidence level and <0.05 significance level. All statistical analyses were performed using the Statistical Package for the Social Sciences (SPSS) version 28.0 (SPSS Inc., Chicago, IL, USA, 2020) [33].

## 3. Results

### 3.1. Sample Characteristics

Out of 591 included participants, 67.7% were females, 56.7% were aged 22 years old or less, 68.2% held Czech nationality, and 61.8% followed the RUW-22 news at least once daily. The most frequent news outlet was digital news portals (82.8%), followed by social media networks (72.4%) and television (37.5%) (Table S1).

Female (7.51 ± 2.24), >22 years old (7.42 ± 2.49) and Slovak students (7.68 ± 2.04) had significantly higher levels of “feeling concerned with the RUW-22 news” compared with their male (6.45 ± 2.88), ≤22 years old (7.00 ± 2.50) and Czech counterparts (7.05 ± 2.56). The level of “feeling concerned” was significantly associated with news-following frequency (*Sig*. < 0.001), as the students who reported following the news every couple of hours had the highest level of “feeling concerned” (8.73 ± 1.64) while the students who reported not following the news had the lowest level (4.71 ± 3.27).

### 3.2. Generalized Anxiety and Depressive Symptoms

Overall, the mean GAD-7 score of our participants was 7.86 ± 5.32 (0–21); their mean PHQ-9 score was 8.66 ± 6.29 (0–27). Given the GAD-7 scores, 31.6%, 32.3%, 22.3%, and 13.7% of the participants exhibited minimal, mild, moderate, and severe anxiety symptoms, respectively. Given the PHQ-9 scores, 31.5%, 28.4%, 22%, 11%, and 7.1% of the participants exhibited none-minimal, mild, moderate, moderately severe, ad severe depressive symptoms, respectively.

Females had significantly higher GAD-7 and PHQ-9 scores (8.64 and 9.38) than their male peers (6.11 and 7.03), respectively. No significant differences in GAD-7 or PHQ-9 scores were found among age groups or nationalities. The highest GAQ-7 and PHQ-9 scores were reported for the participants who followed the news every couple of hours (11.03 and 12.40), while those who reported the lowest scores were not following the news at all (6.50 and 7.38). Social media networks were associated with the highest GAD-7 and PHQ-9 scores (8.38 and 9.14) (Table S1).

### 3.3. Civilian Uses of Nuclear Power

While 64.5% of the participants agreed that nuclear power was safe and 79.7% denied being afraid that nuclear power plants could deteriorate their health, 56.9% thought that public acceptance was important for constructing new nuclear power plants in their countries. The GAD-7 score was significantly (*Sig*. = 0.031) associated with the item “I think that nuclear power is safe”, as those who disagreed with this notion had the highest GAD-7 scores. The participants who did not think public acceptance was important for new nuclear power plant establishment had the lowest “feeling concerned” levels. PHQ-9 scores were not significantly associated with any of the civilian usage items (Table 1).

### 3.4. Nuclear War-Related Anxiety

About 42.1% and 45.5% of the participants reported feeling depressed at the possibility of nuclear war and agreed that the chances that there would be a nuclear war in their lifetime were very high, respectively. In addition, more than two-thirds (64.5%) agreed with the notion that mankind seemed on a sure track to self-destruction. On the other hand, 67.3% and 56.7% of the participants agreed that eliminating the possibility of a nuclear war, at any price, should be everyone’s highest priority and that nobody should be allowed to advocate building nuclear weapons, respectively. Only 19.8% and 5.9% of the participants agreed that the basic goodness of humanity could rule out the possibilities of a nuclear war and that a limited nuclear war would not have much effect on their lives, respectively.

The level of “feeling concerned” was significantly associated with the notions of nuclear war possibility, as those who felt depressed at the possibility of a nuclear war (8.15 ± 1.82) had significantly the highest levels of “feeling concerned” compared to those who did not feel depressed (6.13 ± 2.84). The participants who agreed with the notions of nuclear weapons elimination and advocacy (7.36 ± 2.37 and 7.54 ± 2.26) had significantly higher levels of “feeling concerned” compared to those who did not agree with these notions (6.11 ± 3.01 and 6.16 ± 3.05) (Table 2).

The mean GAD-7 and PHQ-9 scores were significantly higher among those who felt depressed at the possibility of a nuclear war (9.81 ± 5.17 and 10.19 ± 6.31), agreed that the chances that there would be a nuclear war in their lifetime were very high (8.93 ± 5.41 and 9.94 ± 6.43), and agreed that the mankind seemed to be on a sure track to self-destruction (8.33 ± 5.60 and 9.41 ± 6.59) compared with those who did not feel depressed (6.10 ± 5.14 and 7.14 ± 5.97), did not agree the nuclear war chances were high (6.45 ± 4.80 and 7.16 ± 5.87), and did not agree that mankind seemed to be on a sure track to self-destruction (6.37 ± 4.44 and 6.58 ± 5.03) (Table 2).

Regarding the statement “I often feel depressed at the possibility of a nuclear war”, female participants were more likely (*Sig*. < 0.001) to agree with it than males (49.8% vs. 28.2%, respectively). No statistically significant differences were found among different age groups or nationalities. Moreover, the students who had a higher level of “feeling concerned” were more likely (*Sig*. < 0.001) to agree with this statement (56.2% vs. 25.6%, respectively). Similarly, the participants who followed the news every couple of hours were more likely (*Sig*. = 0.025) to agree with this statement compared with those who did not follow the news (57.1% vs. 26.5%, respectively) (Table S2).

**Figure 1:**
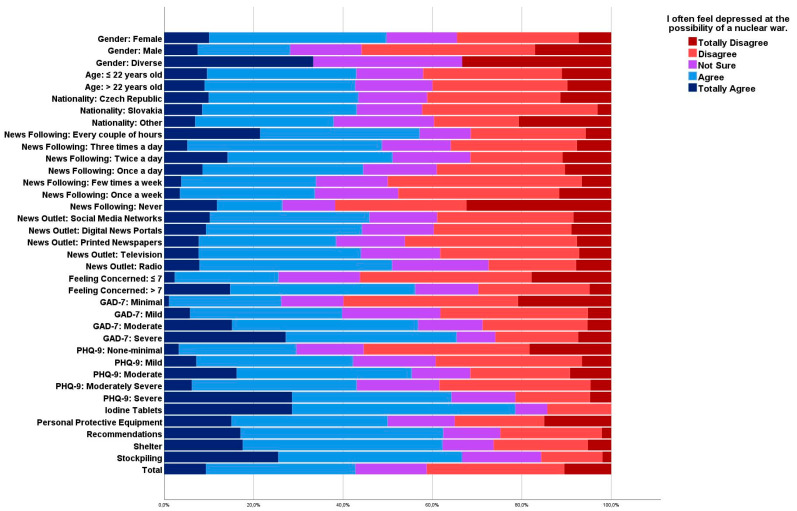
Response to “I often feel depressed at the possibility of a nuclear war” across various independent variables, April–May 2022, (*n* = 591).

No statistically significant differences were found among news outlets. The higher levels of GAD-7 and PHQ-9 scores were significantly associated with a higher likelihood of agreement (*Sig*. < 0.001) (Figure 1).

### 3.5. Preparedness Measures

When asked about their preparedness measures during the previous four weeks, less than one quarter (23.9%) reported looking for recommendations for protection against nuclear accidents, and less than one-fifth (19.3%) were looking for the nearest bomb shelter. Additionally, 8.6%, 3.4%, and 2.4% of the participants reported stockpiling food, fuel or other essential products, purchasing personal protective equipment (PPE), and purchasing iodine tablets, respectively (Table 3).

The participants who were looking for recommendations for protection against nuclear accidents had significantly (*Sig*. < 0.001) higher levels of “feeling concerned” (8.16 vs. 6.87), GAD-7 score (9.46 vs. 7.36), and PHQ-9 score (10.70 vs. 8.02). Similarly, the participants who were looking for the nearest bomb shelter had significantly (*Sig*. < 0.001) higher levels of “feeling concerned” (7.97 vs. 6.98), GAD-7 score (9.54 vs. 7.46), and PHQ-9 score (9.83 vs. 8.38). Stockpiling was significantly (*Sig*. < 0.001) associated with higher levels of GAD-7 (11 vs. 7.56) and PHQ-9 (12.39 vs. 8.31) scores (Table 3).

### 3.6. Correlation Analysis

The non-parametric correlation was carried out as the numerical variables were not normally distributed according to the Shapiro–Wilk test. The level of “feeling concerned” was positively and relatively strongly correlated with the news-following frequency (*r_s_* = 0.445), GAD-7 score (*r_s_* = 0.454), and depression about nuclear war possibility (*r_s_* = 0.401). It was also positively but moderately correlated with the PHQ-9 score (*r_s_* = 0.326). The scores of GAD-7 and PHQ-9 were strongly correlated (*r_s_* = 0.764). The depression about nuclear war possibility was positively yet moderately correlated with GAD-7 (*r_s_* = 0.377) and PHQ-9 (*r_s_* = 0.274) scores (Table 4).

### 3.7. Regression Analysis

The linear regression model for “feeling depressed at the possibility of a nuclear war” as an outcome variable had an adjusted r-squared value of 25.4%. The severe and moderate levels of GAD-7 had beta values of 0.49 (95% CI: 0.17–0.81) and 0.53 (0.14–0.92), respectively. Similarly, feeling concerned about the war had a beta value of 0.16 (0.12–0.20). Regarding precautions, stockpiling and searching for recommendations for nuclear accidents had beta values of 0.51 (0.19–0.83) and 0.27 (0.05–0.50), respectively (Table 5).

## 4. Discussion

The current study found that nearly half of our sample of Czech university students were concerned about the outbreak of a nuclear accident due to the Russian–Ukrainian War 2022 (RUW-22) and the potential consequences of this war on their lives. Nearly a quarter of the participants started preparing for the possibility of a nuclear accident by undertaking specific measures such as learning protection measures against nuclear accidents, looking for the nearest bomb shelters, stockpiling food, fuel or other essential products, and purchasing personal protective equipment (PPE). The fear of nuclear war was significantly associated with higher levels of “feeling concerned”, depression (PHQ-9) and anxiety (GAD-7). It was also positively but weakly correlated with the frequency of following the RUW-22-related news that had impacted perceived safety and peace in Europe and the world.

With the breakout of the RUW-22 on 24 February 2022, public concern about the possibility of a nuclear war started to increase, and it reached its peak on the 27 February when the Russian president, Vladimir Putin, ordered his nuclear deterrent forces to be on high alert [15]. The concern about nuclear war was evident in the search terms used on Google, where there was a significant rise in the use of terms such as nuclear war, nuclear weapon, Chernobyl disaster, and bomb shelters [35]. The use of these terms reached a peak on 24 February (the first day of the war), 27 February (when the Russian president put his nuclear forces on high alert), and 4 March (when the Russian troops seized control on the largest nuclear power plant in Europe). Our results also reflect the current increasing fear of nuclear war, as 42.1% of the participants reported feeling depressed at the possibility of nuclear war, and 45.5% agreed that the chances that there would be a nuclear war in their lifetime were very high.

This high rate of concern about the nuclear war among Czech youth may be partly explained by the current war (RUW-22). The association between international crises and the increase in nuclear anxiety was evident in several studies [1,36,37]. As a part of a national survey started in 1975 in the US, 7.5% of senior high school students reported that they are often worried about the possibility of nuclear war. This percentage increased by about four folds in 1980 [37], which coincided with the hostage crisis in Iran (when the US embassy in Iran was attacked and about 70 Americans were held captive) [38]. Another factor that may contribute to this fear is the nuclear history of the region. On 26 April 1986, the region witnessed one of the worst nuclear disasters in history, known as the “Chernobyl disaster”, which happened at the Chernobyl nuclear power plant in Ukraine. This accident impacted the health of hundreds of thousands in Ukraine and other countries in the region [39]. The mental health impact of the Chernobyl disaster exceeded that of physical health and was considered the largest public health problem caused by this disaster, with its effects continuing to the day [39]. The fear of nuclear war was also reported before the Chernobyl disaster. In a study conducted on more than 900 American adolescents (12–19 years) in 1982, 31.9% of the students reported that they felt very worried about nuclear war, while 17.6% reported that they felt very worried about nuclear power leaks [40]. In summary, the fear of nuclear power and the damage it can cause has existed for decades, and it fluctuates with international events (such as the Chernobyl disaster and RUW-22) [1,36,37].

Several studies found that exposure to a nuclear threat is associated with negative mental health outcomes. In reviewing the literature on three major nuclear accidents, the Three Mile Island nuclear accident in the United States (1979), the Chernobyl nuclear disaster in Ukraine (1986), and the Fukushima nuclear disaster in Japan (2011), there were higher levels of PTSD, anxiety, and depression among those who experienced one of those disasters compared to who were not exposed to such a threat [41]. The mental health impact of nuclear accidents is not limited to those directly affected. A study that followed up on a number of high school students in Finland between 1991–1995 found a positive association between the fear of nuclear war and common mental disorders. There were also higher levels of fear of nuclear war among females compared to male participants [42,43]. Our study confirms the association between nuclear anxiety and adverse mental health outcomes, as the mean GAD-7 and PHQ-9 scores were significantly higher among young adults who showed concern about the possibility of nuclear war.

Another important finding was that the frequency of following news about RUW-22 was associated with higher levels of nuclear anxiety and higher scores of GAD-7 and PHQ-9. Likewise, media exposure was found to be associated with negative mental health outcomes (such as PTSD, depression, anxiety, and substance use) in different crisis contexts [44]. The impact of media consumption on mental health was evident during the COVID-19 pandemic, where there was a huge amount of misinformation (infodemic), especially on digital media [45,46,47,48,49,50,51,52]. Torales et al. (2022) reported that exposure to longer hours of COVID-19-related news was associated with higher levels of depressive symptoms in the Paraguayan population (OR: 1.933) [53]. The role of the media is critical as it can extend the traumatic effect of a crisis to those who are not directly affected by it. Prior studies confirmed the association between indirect exposure to mass trauma through media and increased trauma-related psychological symptoms [54,55]. The media can also play a positive role during crises by providing the public with information on protecting their well-being. Interestingly, Orui et al. (2020) found that utilising information from the local government about radiation exposure was associated with lower levels of health anxiety among the population affected by the Fukushima nuclear disaster compared to utilizing digital media to obtain information [15].

Given the recency of the war in Ukraine (one month till the start of the data collection), it is expected that some of the participants in this study might have developed some symptoms as a normal response to the current events, and they will improve over time, Still, some can develop a mental health condition, so it is important to raise awareness among students about mental health problems and when to seek help [12,13].

### 4.1. Strengths

To the best of our knowledge, this study is the first to explore nuclear anxiety in the context of RUW-22 and to evaluate its associated demographic and psychosocial variables among young adults. In our previous article, we focused on the mental health burden of the RUW-22, represented by GAD-7 and PHQ-9 scores for anxiety and depression [17]. All published studies—to date—that were concerned with the mental health aspects of RUW-22 had overlooked the role of nuclear anxiety as a potential trigger/mediator for psychologic disorders during this militant conflict [18,19,20].

The recruited sample reflected the target population’s female/male and national/foreign student ratios [17,56]. Anonymous data collection aimed to reduce the information bias and Hawthorne’s effect. Another value of this study is the use of GAD-7 and PHQ-9, which are widely accepted and can yield internationally comparable results.

### 4.2. Limitations

The present study has several limitations. First, using a non-random recruitment strategy inevitably leads to a biased sample. Second, the cross-sectional design does not facilitate the evaluation of trends in nuclear anxiety and common psychological disorders. Third, the prevalence and severity of anxiety and depressive symptoms could have been overestimated due to self-selection bias that could not be omitted in this study design. Fourth, the findings of this study should be considered as an immediate response to the RUW-22, while most psychological disorders, including depression, typically take longer intervals. Therefore, future studies are needed to re-evaluate the situation in medium and long term.

### 4.3. Implications

The mental health impact of wars and conflicts exceeds the borders of the conflicting countries. Hence, governments, international organisations, non-governmental organisations, and other stakeholders should consider the short-term and long-term impacts of the RUW-22 on their youth’s mental health. The media plays a vital role during disasters, and how they display disaster-related news might impact the public’s mental health. Mass media can play a positive role during disasters by informing the public on how to reduce risk and protect their well-being.

## 5. Conclusions

Within the limitations of the present study, nuclear anxiety was found to be common among Czech university students. Its associated factors may include, but are not limited to, the female gender, common psychological disorders such as generalised anxiety and depression, RUW-22-related news-following frequency, and the level of “feeling concerned”.

## Figures and Tables

**Table 1:** Attitudes towards Civilian Usages of Nuclear Power among Czech Universities Students Participating in the RUW-22 Survey, April–May 2022, (*n* = 591).

Variable	Outcome	Frequency (%)	Feeling Concerned	*Sig.*	GAD—7	*Sig.*	PHQ—9	*Sig.*
I think that nuclear power is safe.	Totally Disagree	40 (6.8%)	7.32 ± 2.35	0.543	9.35 ± 5.43	0.031	9.87 ± 6.92	0.238
	Disagree	50 (8.5%)	7.54 ± 1.81		8.50 ± 4.41		9.34 ± 5.91	
	Not Sure	120 (20.3%)	7.04 ± 2.54		8.44 ± 5.39		9.27 ± 6.37	
	Agree	236 (39.9%)	7.42 ± 2.27		7.68 ± 5.27		8.43 ± 6.26	
	Totally Agree	145 (24.5%)	6.72 ± 2.98		7.04 ± 5.49		7.98 ± 6.19	
In my opinion, public acceptance is important for the construction of new nuclear power plants.	Totally Disagree	19 (3.2%)	5.84 ± 3.20	0.050	7.79 ± 6.16	0.080	8.89 ± 7.33	0.143
	Disagree	75 (12.7%)	6.41 ± 3.11		6.99 ± 6.17		7.81 ± 6.92	
	Not Sure	161 (27.2%)	7.25 ± 2.35		8.61 ± 4.94		8.95 ± 6.08	
	Agree	258 (43.7%)	7.49 ± 2.23		7.62 ± 5.11		8.36 ± 6.13	
	Totally Agree	78 (13.2%)	7.01 ± 2.60		7.96 ± 5.59		9.83 ± 6.29	
I am afraid that nuclear power plants in my country can deteriorate my health condition even if they are working in normal operation mode.	Totally Disagree	274 (46.4%)	7.00 ± 2.77	0.312	7.53 ± 5.55	0.205	8.34 ± 6.33	0.596
	Disagree	197 (33.3%)	7.44 ± 2.17		7.79 ± 5.06		8.80 ± 6.21	
	Not Sure	81 (13.7%)	6.91 ± 2.31		8.51 ± 4.89		8.89 ± 5.78	
	Agree	27 (4.6%)	7.37 ± 2.62		9.07 ± 5.74		9.26 ± 7.32	
	Totally Agree	12 (2.0%)	8.08 ± 1.56		9.42 ± 5.70		11.00 ± 7.78	

One Sample Proportion test and Kruskal–Wallis test (*H*) had been used with a significance level (*Sig*.) ≤ 0.05.

**Table 2:** Nuclear War-Related Anxiety among Czech Universities Students Participating in the RUW-22 Survey, April–May 2022, (*n* = 591).

Variable	Outcome	Frequency (%)	Feeling Concerned	*Sig.*	GAD—7	*Sig.*	PHQ—9	*Sig.*
I often feel depressed at the possibility of a nuclear war.	Totally Disagree	62 (10.5%)	4.40 ± 3.34	<0.001	4.69 ± 5.46	<0.001	5.73 ± 5.97	<0.001
	Disagree	182 (30.8%)	6.71 ± 2.39		6.58 ± 4.95		7.63 ± 5.91	
	Not Sure	94 (15.9%)	7.28 ± 2.05		7.18 ± 4.38		8.51 ± 6.12	
	Agree	194 (33.5%)	7.93 ± 1.90		8.87 ± 4.93		9.40 ± 5.98	
	Totally Agree	55 (9.3%)	8.93 ± 1.20		13.18 ± 4.59		13.02 ± 6.71	
The chances that there will be a nuclear war in my lifetime are very high.	Totally Disagree	22 (3.7%)	4.68 ± 3.47	<0.001	4.68 ± 5.03	<0.001	5.18 ± 4.04	<0.001
	Disagree	110 (18.6%)	6.71 ± 2.51		6.81 ± 4.70		7.55 ± 6.10	
	Not Sure	190 (32.1%)	6.96 ± 2.61		7.32 ± 5.24		7.91 ± 6.04	
	Agree	216 (36.5%)	7.51 ± 2.13		8.31 ± 5.10		9.40 ± 6.22	
	Totally Agree	53 (9.0%)	8.55 ± 1.90		11.49 ± 5.92		12.11 ± 6.88	
Mankind seems on a sure track to self-destruction.	Totally Disagree	24 (4.1%)	6.00 ± 3.43	0.008	5.08 ± 3.60	0.009	4.46 ± 3.38	<0.001
	Disagree	75 (12.7%)	6.93 ± 2.52		6.79 ± 4.62		7.25 ± 5.29	
	Not Sure	111 (18.8%)	7.63 ± 2.12		7.58 ± 4.78		7.95 ± 5.77	
	Agree	215 (36.4%)	6.87 ± 2.54		7.93 ± 4.46		8.69 ± 6.21	
	Totally Agree	166 (28.1%)	7.54 ± 2.43		8.84 ± 7.76		10.35 ± 6.96	
Eliminating the possibility of a nuclear war, at any price, should be everyone’s highest priority.	Totally Disagree	23 (3.9%)	5.43 ± 3.46	0.001	5.87 ± 4.39	0.426	8.22 ± 6.37	0.701
	Disagree	57 (9.6%)	6.39 ± 2.79		7.98 ± 5.46		9.42 ± 6.88	
	Not Sure	113 (19.1%)	7.26 ± 2.41		7.60 ± 5.14		8.46 ± 6.37	
	Agree	211 (35.7%)	7.13 ± 2.42		7.88 ± 5.22		8.29 ± 6.12	
	Totally Agree	187 (31.6%)	7.63 ± 2.28		8.21 ± 5.58		9.03 ± 6.25	
Nobody should be allowed to advocate, publicly or privately, the building of nuclear weapons.	Totally Disagree	46 (7.8%)	5.54 ± 3.51	<0.001	5.65 ± 5.55	0.004	7.20 ± 6.55	0.168
	Disagree	63 (10.7%)	6.60 ± 2.59		7.06 ± 5.25		8.08 ± 6.02	
	Not Sure	147 (24.9%)	7.10 ± 2.38		7.66 ± 4.96		8.28 ± 5.53	
	Agree	163 (27.6%)	7.32 ± 2.17		7.96 ± 4.97		8.56 ± 5.93	
	Totally Agree	172 (29.1%)	7.74 ± 2.33		8.82 ± 5.71		9.69 ± 7.12	
The basic goodness of humanity rules out any real possibility of nuclear war.	Totally Disagree	147 (24.9%)	7.07 ± 2.74	0.701	7.86 ± 5.59	0.710	8.86 ± 6.56	0.802
	Disagree	178 (30.1%)	7.33 ± 2.18		7.77 ± 5.12		8.44 ± 5.94	
	Not Sure	149 (25.2%)	6.97 ± 2.54		7.58 ± 5.27		8.48 ± 6.27	
	Agree	81 (13.7%)	7.52 ± 2.21		8.04 ± 5.06		8.49 ± 6.29	
	Totally Agree	36 (6.1%)	6.92 ±3.32		9.08 ± 5.99		10.08 ± 7.07	
I do not think that a limited nuclear war would have much effect on my life.	Totally Disagree	308 (52.1%)	7.58 ± 2.29	<0.001	8.36 ± 5.39	0.027	8.98 ± 6.14	0.082
	Disagree	180 (30.5%)	6.93 ± 2.55		7.61 ± 5.18		8.66 ± 6.56	
	Not Sure	68 (11.5%)	6.24 ± 2.73		6.49 ± 5.23		6.96 ± 5.89	
	Agree	28 (4.7%)	6.36 ± 2.73		6.68 ± 4.85		8.36 ± 5.87	
	Totally Agree	7 (1.2%)	8.00 ± 3.70		10.71 ± 5.47		12.57 ± 8.81	

One Sample Proportion test and Kruskal–Wallis test (*H*) had been used with a significance level (*Sig*.) ≤ 0.05.

**Table 3:** Response to “Within the last 4 weeks, have you undertaken any of the following measures?” of Czech Universities Students Participating in the RUW-22 Survey, April–May 2022, (*n* = 591).

Variable	Outcome	Frequency (%)	Feeling Concerned	*Sig.*	GAD—7	*Sig.*	PHQ—9	*Sig.*
Purchasing iodine tablets	Yes	14 (2.4%)	7.93 ± 2.37	0.166	9.79 ± 5.09	0.151	11.93 ± 6.26	0.041
	No	577 (97.6%)	7.16 ± 2.50		7.81 ± 5.32		8.58 ± 6.27	
Purchasing personal protective equipment (PPE)	Yes	20 (3.4%)	7.20 ± 2.49	0.726	8.35 ± 5.76	0.725	10.80 ± 7.76	0.227
	No	571 (96.6%)	7.17 ± 2.86		7.84 ± 5.31		8.59 ± 6.23	
Looking for recommendations for protection against nuclear accidents	Yes	141 (23.9%)	8.16 ± 1.95	<0.001	9.46 ± 5.23	<0.001	10.70 ± 6.60	<0.001
	No	450 (76.1%)	6.87 ± 2.58		7.36 ± 5.25		8.02 ± 6.06	
Looking for the nearest bomb shelter	Yes	114 (19.3%)	7.97 ± 2.14	<0.001	9.54 ± 5.29	<0.001	9.83 ± 6.39	0.019
	No	477 (80.7%)	6.98 ± 2.54		7.46 ± 5.25		8.38 ± 6.24	
Stockpiling food, fuel, or other essential products	Yes	51 (8.6%)	7.53 ± 2.86	0.062	11.00 ± 5.87	<0.001	12.39 ± 7.43	<0.001
	No	540 (91.4%)	7.14 ± 2.46		7.56 ± 5.17		8.31 ± 6.06	

Mann–Whitney test (*U*) was used with a significance level (*Sig*.) ≤ 0.05.

**Table 4:** Correlation Between “Feeling Concerned”, News-Following Frequency, GAD–7, PHQ–9, and Nuclear War Threat among Czech Universities Students Participating in the RUW-22 Survey, April–May 2022, (*n* = 591).

		Feeling Concerned	News Fol. Frequency	GAD–7	PHQ–9	NW Possibility
Feeling Concerned	*rs*	1.000				
	*Sig.*	N/A				
News-Following Frequency	*rs*	0.445	1.000			
	*Sig.*	<0.001	N/A			
GAD–7	*rs*	0.454	0.198	1.000		
	*Sig.*	<0.001	<0.001	N/A		
PHQ–9	*rs*	0.326	0.181	0.764	1.000	
	*Sig.*	<0.001	<0.001	<0.001	N/A	
I often feel depressed at the possibility of a nuclear war.	*rs*	0.401	0.196	0.377	0.274	1.000
	*Sig.*	<0.001	<0.001	<0.001	<0.001	N/A

Spearman’s correlation was used with a significance level (*Sig*.) ≤ 0.05. To interpret Spearman’s correlation coefficient values (*rs*): 0–0.10 (negligible correlation), 0.10–0.19 (weak correlation), 0.20–0.39 (moderate correlation), 0.40–0.59 (relatively strong correlation), 0.60–0.79 (strong correlation), and 0.80–1 (very strong correlation) [34].

**Table 5:** Regression Analysis of “feeling depressed at the possibility of a nuclear war” among Czech Universities Students Participating in the RUW-22 Survey, April–May 2022, (*n* = 591).

Predictor	β	SE	95% CI	*t*	*Sig.*
Intercept *	1.48	0.14	1.21–1.76	10.66	<0.001
GAD-7: Mild vs. Minimal	0.21	0.12	−0.03–0.45	1.70	0.090
GAD-7: Moderate vs. Minimal	0.49	0.16	0.17–0.81	3.03	0.003
GAD-7: Severe vs. Minimal	0.53	0.20	0.14–0.92	2.69	0.007
PHQ-9: Mild vs. None-minimal	0.10	0.12	−0.14–0.33	0.79	0.427
PHQ-9: Moderate vs. None-minimal	0.06	0.15	−0.24–0.36	0.41	0.686
PHQ-9: Moderately Severe vs. None-minimal	−0.31	0.19	−0.68–0.06	−1.63	0.105
PHQ-9: Severe vs. None-minimal	0.12	0.23	−0.32–0.57	0.54	0.587
Feeling Concerned	0.16	0.02	0.12–0.20	7.72	<0.001
News-following frequency	−0.01	0.03	−0.07–0.04	−0.51	0.608
Precautions: Iodine Tables (Yes vs. No)	0.52	0.29	−0.04–1.08	1.79	0.074
Precautions: PPE (Yes vs. No)	−0.15	0.24	−0.63–0.32	−0.63	0.527
Precautions: Recommendations (Yes vs. No)	0.27	0.11	0.05–0.50	2.39	0.017
Precautions: Shelter (Yes vs. No)	0.17	0.12	−0.07–0.40	1.38	0.168
Precautions: Stockpiling (Yes vs. No)	0.51	0.16	0.19–0.83	3.17	0.002

* Represents reference level.

## Data Availability

The data that support the findings of this study are available from the corresponding author upon reasonable request.

## Supplementary Materials

The following supporting information can be downloaded at: https://www.mdpi.com/article/10.3390/ijerph20043551/s1, Table S1: Sample Characteristics of Czech University Students Participating in the RUW-22 Survey, April–May 2022, (*n* = 591); Table S2: Response to “I often feel depressed at the possibility of a nuclear war” across various independent variables, April–May 2022, (*n* = 591).
