# Supplementary material for: Nuclear Anxiety Amid the Russian-Ukrainian War 2022 (RUW-22): Descriptive Cross-Sectional Study
Source: Int J Environ Res Public Health. 2023 Feb 17;20(4):3551. doi: 10.3390/ijerph20043551 (PMC9962827; doi:10.3390/ijerph20043551)
Supplement: Supplementary file 1 [file ijerph-20-03551-s001.zip › ijerph-2118877-supplementary.pdf]

International Journal of Environmental Research and Public Health

MDPI

Table S1. Sample Characteristics of Czech University Students Participating in the RUW-22 Survey, April – May 2022, (n = 591)

Variable	Outcome	Frequency (%)	Feeling Concerned 7.17 ± 2.50 (0 – 10)	*Sig.*	GAD–7 7.86 ± 5.32 (0 – 21)	*Sig.*	PHQ–9 8.66 ± 6.29 (0 – 27)	*Sig.*
Gender	Female	400 (67.7%)	7.51 ± 2.24	**< 0.001**	8.64 ± 5.29	**< 0.001**	9.38 ± 6.41	**< 0.001**
	Male	188 (31.8%)	6.45 ± 2.88		6.11 ± 4.96		7.03 ± 5.68	
	Diverse	3 (0.5%)	7.67 ± 1.53	0.936	13.00 ± 1.00	0.067	15.33 ± 5.13	0.061
Age Group **	≤ 22 years old	335 (56.7%)	7.00 ± 2.50	**0.016**	7.76 ± 5.12	0.798	8.72 ± 6.07	0.503
	> 22 years old	255 (43.1%)	7.42 ± 2.49		8.02 ± 5.57		8.62 ± 6.58	
Nationality	Czech Republic	403 (68.2%)	7.05 ± 2.56	**0.029**	7.95 ± 5.28	0.987	8.70 ± 6.36	0.768
	Slovakia	130 (22%)	7.68 ± 2.04		7.92 ± 5.01		8.68 ± 5.89	
	Other	58 (9.8%)	6.91 ± 2.91	0.743	7.12 ± 6.25	0.113	8.34 ± 6.74	0.536
News Following Frequency	Every couple of hours	70 (11.8%)	8.73 ± 1.64	**< 0.001**	11.03 ± 5.84	**< 0.001**	12.40 ± 7.08	**0.001**
	Three times a day	39 (6.6%)	8.38 ± 2.12		7.85 ± 4.74		8.67 ± 6.12	
	Twice a day	92 (15.6%)	8.03 ± 1.88		8.09 ± 5.13		8.43 ± 6.12	
	Once a day	164 (27.7%)	7.30 ± 2.32		7.82 ± 5.14		9.01 ± 6.18	
	Few times a week	106 (17.9%)	6.61 ± 2.21		7.03 ± 4.94		7.39 ± 5.92	
	Once a week	86 (14.6%)	5.86 ± 2.62		6.70 ± 5.18		7.29 ± 5.46	
	Never	34 (5.8%)	4.71 ± 3.27		6.50 ± 5.42		7.38 ± 6.05	
Frequently Used News Outlets	Social Media Networks	403 (72.4%)	7.61 ± 2.19	**< 0.001**	8.38 ± 5.32	**0.001**	9.14 ± 6.29	**0.007**
	Digital News Portals	461 (82.8%)	7.38 ± 2.34	0.301	7.89 ± 5.33	0.512	8.62 ± 6.21	0.397
	Printed Newspapers	13 (2.3%)	7.69 ± 1.65	0.873	6.31 ± 6.07	0.181	6.62 ± 6.38	0.136
	Television	209 (37.5%)	7.53 ± 2.12	0.301	7.99 ± 5.21	0.828	8.65 ± 6.17	0.858
	Radio	51 (9.2%)	7.65 ± 2.36	0.262	7.59 ± 4.77	0.698	8.27 ± 5.66	0.769

* Mann–Whitney test (U) and Kruskal–Wallis test (H) were used with a significance level (*Sig.*) ≤ 0.05.
** One case was missed.

Table S2. Response to "I often feel depressed at the possibility of a nuclear war" across various independent variables, April – May 2022, (n = 591)

Variable	Outcome	Totally Disagree (= 1)	Disagree (= 2)	Not Sure (= 3)	Agree (= 4)	Totally Agree (= 5)	Total (3 ± 1.20)	*Sig.*
Gender	Female	29 (46.8%)	109 (59.9%)	63 (67%)	159 (80.3%)	40 (72.7%)	3.18 ± 1.15	**<0.001**
	Male	32 (51.6%)	73 (40.1%)	30 (31.9%)	39 (19.7%)	14 (25.5%)	2.63 ± 1.20	
	Diverse	1 (1.6%)	0 (0%)	1 (1.1%)	0 (0%)	1 (1.8%)	3 ± 2	0.997
Age Group **	≤ 22 years old	37 (59.7%)	104 (57.5%)	50 (53.2%)	112 (56.6%)	32 (58.2%)	2.99 ± 1.21	0.806
	> 22 years old	25 (40.3%)	77 (42.5%)	44 (46.8%)	86 (43.4%)	23 (41.8%)	3.02 ± 1.18	
Nationality	Czech Republic	46 (74.2%)	120 (65.9%)	62 (66%)	135 (68.2%)	40 (72.7%)	3.01 ± 1.22	0.705
	Slovakia	4 (6.5%)	51 (28%)	19 (20.2%)	45 (22.7%)	11 (20%)	3.06 ± 1.10	
	Other	12 (19.4%)	11 (6%)	13 (13.8%)	18 (9.1%)	4 (7.3%)	2.84 ± 1.27	0.321
News Following Frequency	Every couple of hours	4 (6.5%)	18 (9.9%)	8 (8.5%)	25 (12.6%)	15 (27.3%)	3.41 ± 1.40	**<0.001**
	Three times a day	3 (4.8%)	11 (6%)	6 (6.4%)	17 (8.6%)	2 (3.6%)	3.10 ± 1.11	
	Twice a day	10 (16.1%)	19 (10.4%)	16 (17%)	34 (17.2%)	13 (23.6%)	3.23 ± 1.06	
	Once a day	17 (27.4%)	47 (25.8%)	27 (28.7%)	59 (29.8%)	14 (25.5%)	3.04 ± 1.19	
	Few times a week	7 (11.3%)	46 (25.3%)	17 (18.1%)	32 (16.2%)	4 (7.3%)	2.81 ± 1.24	
	Once a week	10 (16.1%)	31 (17%)	16 (17%)	26 (13.1%)	3 (5.5%)	2.78 ± 1.12	
	Never	11 (17.7%)	10 (5.5%)	4 (4.3%)	5 (2.5%)	4 (7.3%)	2.44 ± 1.25	
	Social Media Networks	34 (66.7%)	123 (71.5%)	61 (67.8%)	144 (74.6%)	41 (80.4%)	3.09 ± 1.18	0.113
	Digital News Portals	41 (80.4%)	142 (82.6%)	74 (82.2%)	161 (83.4%)	43 (84.3%)	3.05 ± 1.18	0.598

Frequently Used News Outlets	Printed Newspapers	1 (2%)	5 (2.9%)	2 (2.2%)	4 (2.1%)	1 (2%)	2.92 ± 1.19	0.711
	Television	15 (29.4%)	65 (37.8%)	37 (41.1%)	76 (39.4%)	16 (31.4%)	3.06 ± 1.13	0.724
	Radio	4 (7.8%)	10 (5.8%)	11 (12.2%)	22 (11.4%)	4 (7.8%)	3.24 ± 1.11	0.199

* Mann–Whitney test (*U*) and Kruskal–Wallis test (*H*) were used with a significance level (*Sig.*) ≤ 0.05.

** One case was missed.
